# Uterine transposition for fertility preservation prior to pelvic irradiation: an initial Egyptian case series

**DOI:** 10.1186/s43046-026-00395-2

**Published:** 2026-08-11

**Authors:** Basel Refky, Mohamed Abdelkhalek, Mohamed Hamdy, Khalid Atallah, Amr Abuzenah, Amira Zaidan, Sahar Elhawari, Basma Gadelhak, Alaa Ismail, Hisham Ahmed Abou-Taleb, Khaled Gaballa

**Affiliations:** 1 Surgical Oncology Department, Oncology Center, Mansoura University, Al Mansurah, Egypt; 2 Surgical Oncology Department, Bahrain Cancer Center, Bahrain Defence Force Royal Medical Services, Ar Rifā‘, Bahrain; 3 Department of Obstetrics and Gynaecology, Al-AHLI Hospital, Hebron, Occupied Palestinian Territory; 4 Department of Obstetrics and Gynaecology, Salmaniya Medical Complex, Manama, Bahrain; 5 Department of Obstetrics and Gynaecology, Faculty of Medicine, University of Khartoum, Khartoum, Sudan; 6 Radiology Department, Faculty of Medicine, Mansoura University, Al Mansurah, Egypt; 7 Department of Obstetrics and Gynaecology, Faculty of Medicine, Assiut University, Assiut, Egypt

**Keywords:** Fertility preservation, Pelvic irradiation, Uterine transposition

## Abstract

**Background:**

Recently, uterine transposition has been proposed as a novel technique for fertility preservation in young females undergoing radiation therapy for pelvic malignancies.

**Patients and methods:**

This is an interventional pilot study including six female patients with pelvic malignancies planned to receive pelvic radiation aiming at preserving their fertility after a multidisciplinary team decision to have uterine transposition. Our study aims to assess the feasibility of this novel technique.

**Results:**

Six patients underwent uterine transposition. Three of them were done through open technique, with successful repositioning. The other three patients underwent laparoscopic uterine transposition, one of them underwent repositioning and still receiving chemotherapy for Ewing’s sarcoma, the other two patients are still receiving radiation therapy after transposition. Unfortunately, one of them died of disease and adjuvant therapy complications four months after laparoscopic uterine transposition.

**Conclusion:**

Uterine transposition is a feasible procedure with safe outcomes. The preliminary results carry a promising hope for better fertility preservation in young patients undergoing radiation for pelvic malignancies.

## Background

The need for Fertility preservation in young females has been increasing in recent decades especially with the advancement of cancer treatments and increasing the disease-free survival and overall survival in variable types of cancer [1]. Several types of pelvic malignancies will require radiation therapy either alone or combined with chemotherapy such as colorectal cancers and pelvic sarcomas. The radiation doses needed in such patients will surely affect their ovarian reserve as well as their uterine integrity by myometrial fibrosis, vascular damage and endometrial injuries [2]. Ovarian transposition was first proposed as a solution to rescue the ovaries from radiation doses in such patients but still the uterus remained in the field of radiation which will hinder future normal pregnancies [3]. In 2017 Ribeiro et al. first described a novel technique for fertility preservation through uterine transposition along with the ovaries in a patient with rectal cancer and since then the technique emerged as an alternative for fertility preservation in such patients [4]. Several authors since then published their experience with these techniques in patients with rectal cancer, gynecologic malignancies as cervical cancer and pelvic sarcomas [5–9]. The first live birth following uterine transposition was documented in 2022 [10]. In this article we will share our experience with this technique in 5 patients who underwent uterine transposition for pelvic malignancies and their outcomes at our institute.

## Patients and methods

This is an interventional pilot study including female patients with pelvic malignancies planned to receive pelvic radiation aiming at preserving their fertility after a multidisciplinary team decision to have uterine transposition, following all the necessary required surgical and medical protocols for follow up. This study aims to assess the feasibility of this novel technique.

The inclusion criteria included women of reproductive age diagnosed with pelvic malignancy, requiring pelvic radiotherapy with accepted ovarian reserve as assessed by anti-mullerian hormone (AMH) and willing to preserve fertility who accepted the surgical procedures after thorough counselling about the predicted outcomes and the possible side effects. The exclusion criteria included patients who have distant metastasis, patients with severe medical co-morbidities and patients above the age of 40 with poor ovarian reserve. All patients received GnRH agonist injections at least a month prior to surgery to stop menstruation. All patients were followed during the first 2 days by vital signs, CBC, CRP and doppler US. Within one month of the operation a follow-up pelviabdominal MRI will be done. Patients who underwent repositioning will also have a pelvic MRI within one month after the operation and CT angiography 3 months after repositioning. For the following 24 months the patients will be followed up after every 6 months as regards the oncological outcome and the ovarian and uterine function by radiology, AMH and the presence of regular menses. This study was performed in line with the principles of the Helsinki declaration. Ethical approval from the Mansoura Faculty of Medicine institutional review board (MFM-IRB) was acquired under the code R.24.07.2702. 

## Results

**Fig. 1:**
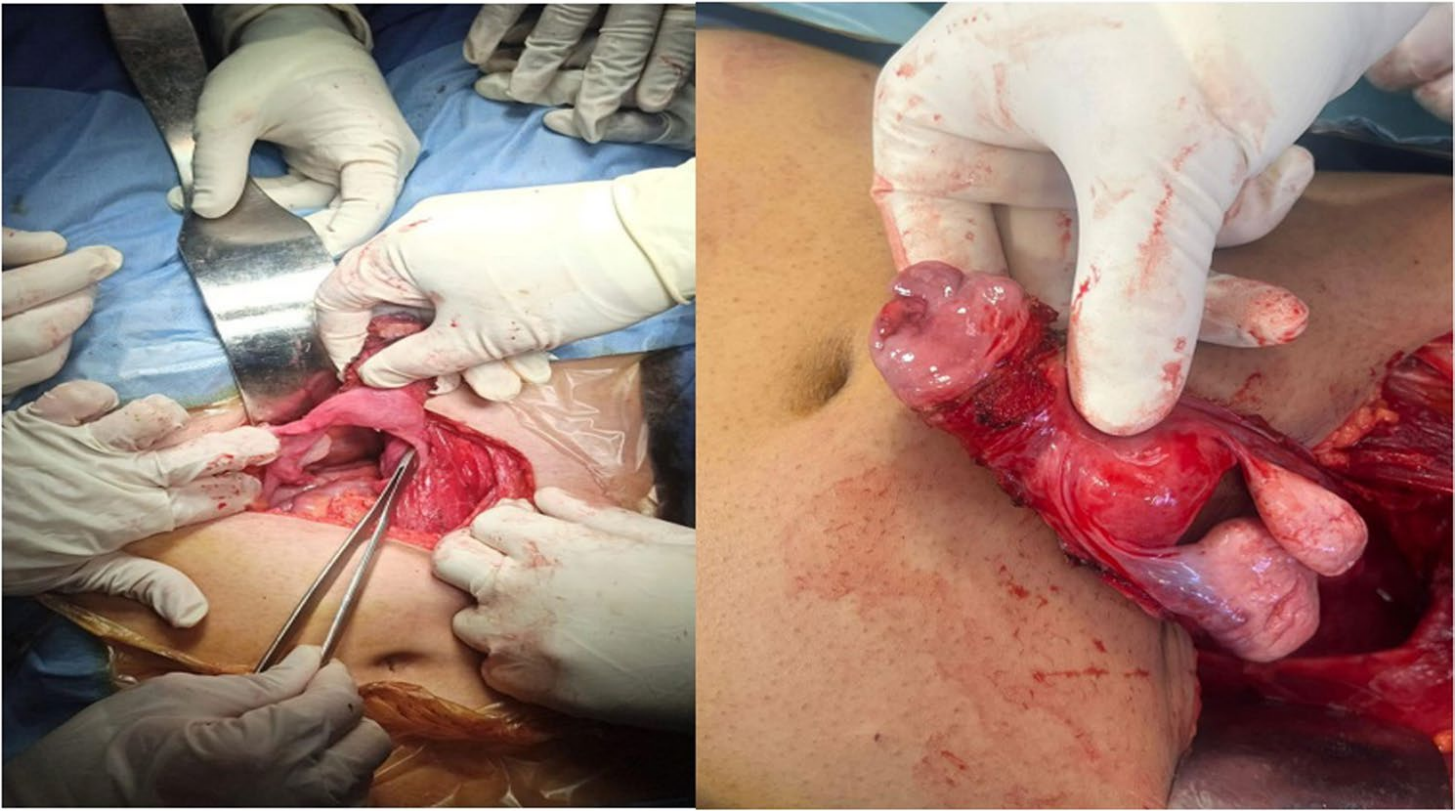
open uterine transposition. The uterus was separated from the vagina and maintained on the ovarian vascular pedicles via the infundibulopelvic ligaments

**Fig. 2:**
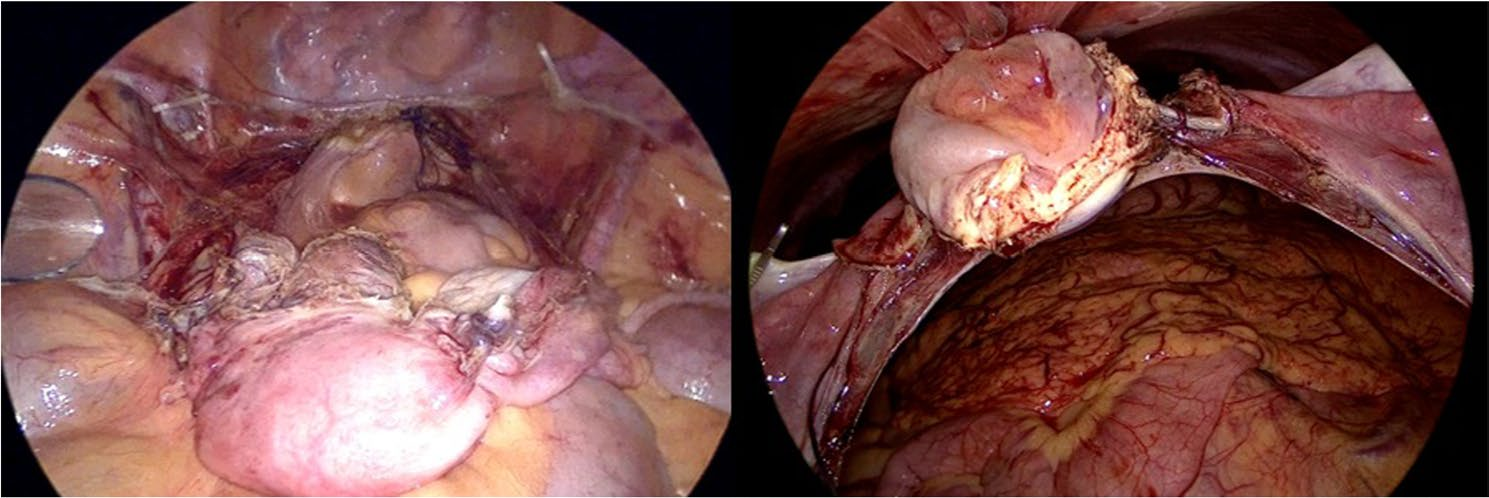
Laparoscopic view of uterus after separation from the vagina then transposition below anterior abdominal wall. Metallic clips on right ovary for marking can be seen in the right picture

**Fig. 3:**
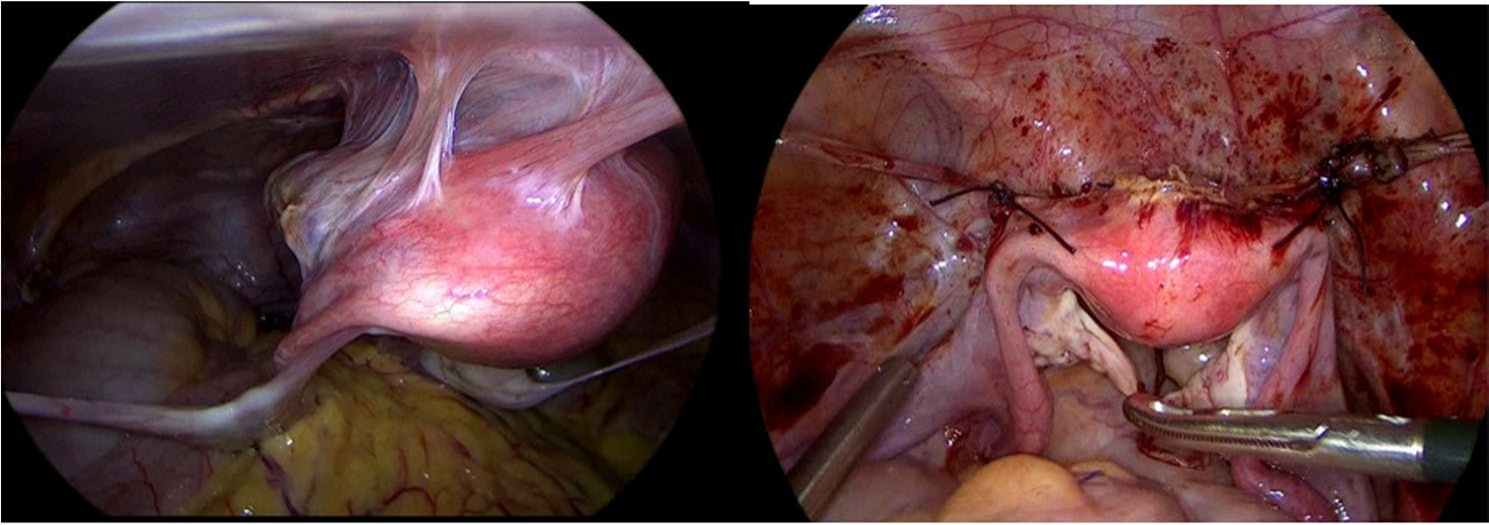
Laparoscopic view of the transpositioned uterus at the beginning of the repositioning operation and adhesions are noted between the uterus and the anterior abdominal wall then at the end of the operation after repositioning in the pelvis

Six patients underwent uterine transposition. 3 patients (50%) underwent uterine transposition through open technique, and all had been repositioned successfully (Fig. 1). 3 patients (50%) underwent laparoscopic uterine transposition (Fig. 2), one of them underwent repositioning and still undergoing chemotherapy for Ewing’s sarcoma (Fig. 3), the other 2 patients (33.3%) are still undergoing radiation therapy after transposition. Unfortunately, one of them died of disease and adjuvant therapy complications 4 months after laparoscopic uterine transposition. Patients’ clinical characteristics are summarized in Table 1.

**Table 1:** Individual patient characteristics and outcomes following uterine transposition

Case	Age (years)	Marital status	Tumor type	Tumor Stage	Surgical approach	OT	Blood loss	complication	Oncological outcome	Hormonal profile
1	18	Single	Left sacro-iliac bone Ewing’s Sarcoma	I	Laparoscopy	**2 h 30 min**	20**cc**	No	NED	Not done
2	18	Single	Left Sacral Ewing’s sarcoma causing left lower limb paralysis	II	Laparoscopy	**3 h**	150cc	No	Died of disease	Not done
3	15	Single	Left Vertebral L5-S1 Ewing’s Sarcoma	I	laparotomy	**1 h**	50cc	No	NED	Not done
4	25	Married	)Malignant Neoplasm of the cervix uteri (Rhabdomyosarcoma	I	laparotomy	**2 h 30 min**	200cc	Yes (fever)	NED	Normal
5	25	Single	Colon cancer	P3TN1M0	laparotomy	**20 min**	150cc	No	NED	Normal
6	25	Married	Rectal cancer	T2 N1 Mx	Laparoscopy	**3 h 30 min**	30cc	No	NED	Normal

*NED No evidence of disease

Regarding the operative outcomes, none of our patients had intra or postoperative complication except one patient (16.6%) who had early postoperative fever and was managed by antipyretics. Doppler US and postoperative MRI showed adequate vascularity of the transpositioned uterus in this patient.

**Fig. 4:**
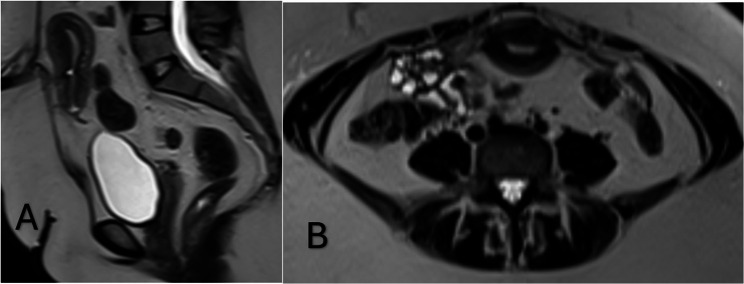
Sagittal (**A**) and Axial (**B**) T2W MRI showing the transpositioned uterus beneath the anterior abdominal wall at the level of the umbilicus with preserved zonal anatomy. Smooth vaginal stump. The transposed right ovary is also noted with follicular activity

**Fig. 5:**
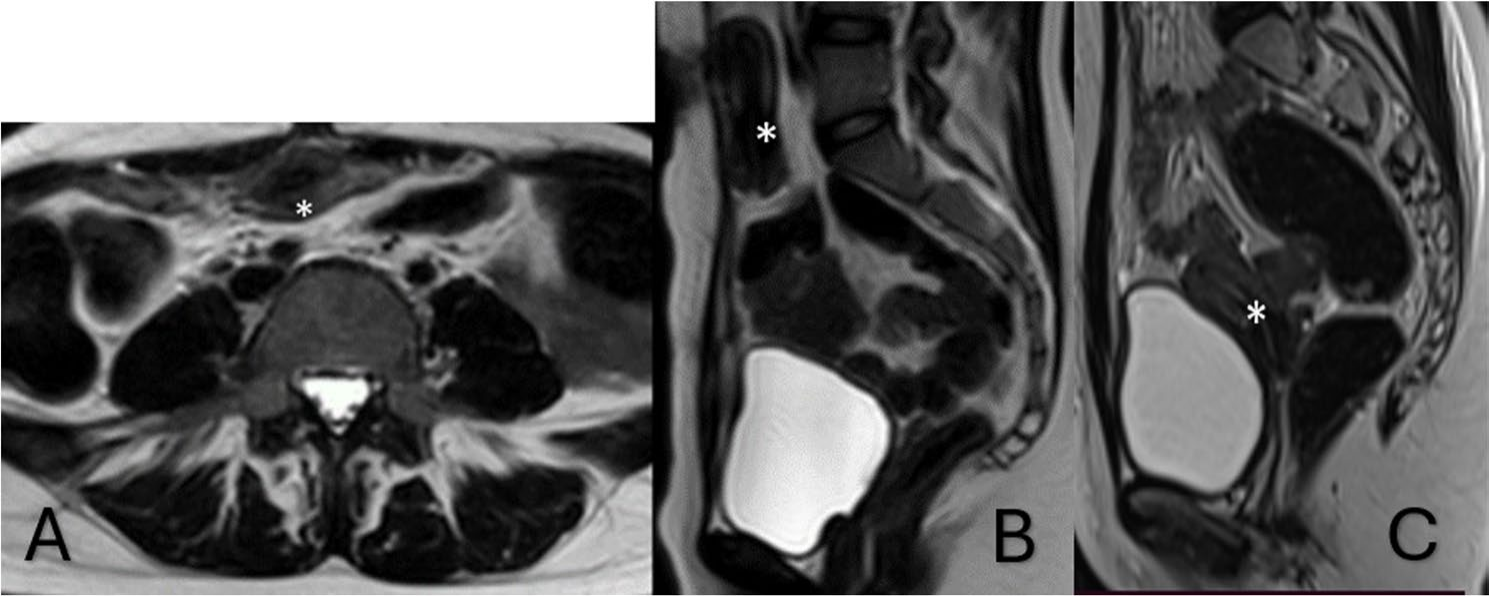
Axial (**A**) and Sagittal (**B**) T2W MRI after uterine transposition. Sagittal T2W MRI (C) then after repositioning of the uterus back into the pelvis. Uterine position is marked by asterisks

All 6 patients underwent postoperative MRI within one month after transposition, in all of which the uterus showed adequate vascularity with preserved zonal differentiation where the lower cervical border at lower edge of L5 (Fig. 4). Ovaries were positioned between level L4 and L5. 4 patients (66.6%) who underwent repositioning also had postoperative MRI showed an adequately located repositioned uterus with preserved zonal anatomy and post contrast enhancement denoting adequate vascularity (Fig. 5). 2 patients (33.3%) who underwent repositioning underwent CT angiography 3 months after repositioning that showed well preserved uterus with neovascularization.

Three patients (50%) who finished their therapy regained normal menses and had normal hormonal profile after repositioning. 2 other patients (33.3%) still under treatment and receiving GnRH Agonists and last patient died from the disease and adjuvant therapy complications 4 months after doing laparoscopic uterine transposition.

## Discussion

Preserving fertility in young patients demanding pelvic radiation for oncological reasons is increasing worldwide even in developing countries. Cryopreservation of oocytes and embryos had been used in these patients with ovarian transposition [11]. However, in these patients the uterus is still exposed to high doses of radiation which hinder future successful pregnancy [2]. Surrogate uterus had been proposed as a solution in this case, but this may not be accepted in certain societies due to religious causes as in ours. Uterine transposition is still an evolving promising technique with limited number of cases done all over the world with about 47 cases have been reported in the literature [9]. Uterine transposition appears to be a suitable technique in this type of patient as it combines preservation of both ovarian and uterine functions.

Regarding the preservation of hormonal function after pelvic radiation, a recent meta-analysis demonstrated preservation of hormonal function in about 66.9% of patients with anal and rectal cancer who had ovarian transposition [12]. Sioulas et al. reported ovarian function preservation in about 90% of patients with non-gynecologic cancers receiving pelvic irradiation with ovaries placed above the level of L5-S1 [13]. As it appears that the higher the level of the ovaries away from radiation field the better hormonal function preservation is achieved. In our study where the uterine cervix is located nearly at level of L5 prior to radiation, the ovaries are at much higher level at left and right hypochondrium opposite L4-L5 reducing the exposure to radiation doses. We documented normal hormonal function in our patients who underwent repositioning after finishing their therapy. Other patients who are still on GnRH agonist will be followed up after finishing their therapy as they are administered GnRH agonist during chemotherapy to prevent premature ovarian insufficiency [14].

Regarding normal menstruation cycles after repositioning a review published by Ribeiro et al. documented 14 out of 16 (87.5%) patients regained normal menses [15]. In our study the 3 patients (100%) who underwent repositioning and finished their chemoradiotherapy regained normal menses.

Regarding the pregnancy rates after uterine transposition. The first live birth after uterine transposition was documented in January 2022 [10]. Lopez et al. reported another successful pregnancy with cesarean section delivery later same year [16]. In a review about uterine transposition out of 15 patients Ribeiro et al. reported 3 successful pregnancies in 3 out of 5 patients who tried to conceive [15]. In a later review by Apelian et al. 4 full term spontaneous pregnancies have been documented [9]. In our study still none of our patients got pregnant as 4 (66.6%) of our patients are still virgins, one of them died of disease, one is divorced and the last one is still undergoing chemoradiotherapy therapy for rectal cancer.

Cervical ischemia and stenosis had been the most common complication after uterine transposition reported in about 28% of cases [15]. In our patients we did not encounter this complication, but this may be attributed to the fact that the clinical assessment of cervical stenosis was limited to 4 out of 6 patients who were still virgins and unmarried, as cultural norms in our society preclude invasive vaginal speculum examination in this demographic. However, all the postoperative radiology did not demonstrate the presence of cervical ischemia.

Regarding the oncological outcome, in a review of 18 cases published in the literature, one patient with rectal cancer died of the disease 4 months after transposition [15]. In another review with median follow up 21.5 months 37 out of 39 patients showed no evidence of recurrence (94.9%) [9] In our study one patient (16.6%) with left sacral Ewing’s sarcoma died of the disease 4 months after doing transposition.

One of the main difficulties that we had in our study is that most of our patients had a long period between transposition and reposition up to six months mainly due to logistics related to radiation therapy as there is limited number of radiotherapy machines in our locality, so the patients usually suffer some delays in their treatment schedule. No definite time was reported in the literature between the 2 surgeries but usually reposition was done after completing the radiation sessions which usually takes about 6 to 8 weeks [17, 18].

In our study the surgical approach either by laparoscopy or laparotomy was mainly guided by surgeon preference as it is a new technique with a limited number of cases worldwide and still there is no guidelines for standardization of the approach. Also, in our study we did not compare the outcomes between the two approaches due to the limited number of cases and the study was not designed to address the advantages and disadvantages of both approaches in uterine transposition.

In our study we had some limitations one of them is that not all the patients had finished their therapy, and we often experience delays in therapy schedules in our country due to limited number of radiotherapy machines and the presence of relatively long waiting lists. Also, not all patients who underwent uterine transposition have undergone repositioning yet. One of the strength points of this study is that all the data is prospective although it is not a randomized controlled trial, but this is due to the relative rarity and novelty of this technique and the small number of patients. To our knowledge the number of cases reported worldwide is still less than 50 cases. In this study we report 6 cases done at the same institute. The results of our study carry a promising hope for fertility preservation in young patients undergoing pelvic radiation. We encourage future multicenter prospective randomized controlled trials for better pooling of larger number of patients to demonstrate more information about the procedure, patient selection, benefits and drawbacks.

## Conclusion

Uterine transposition seems to be a feasible procedure with safe outcomes in experienced hands. The preliminary results carry a promising hope for better fertility preservation in young patients undergoing radiation for pelvic malignancies.

## Data Availability

All data supporting the findings of this study are available within the paper. The datasets used and/or analyzed during the current study are available from the corresponding author on reasonable request.
